# A Framework for a Context-Aware Elderly Entertainment Support System

**DOI:** 10.3390/s140610538

**Published:** 2014-06-16

**Authors:** M. Anwar Hossain, Atif Alamri, Ahmad S. Almogren, SK Alamgir Hossain, Jorge Parra

**Affiliations:** 1 College of Computer and Information Sciences (CCIS), King Saud University, 11543 Riyadh, Saudi Arabia; E-Mails: atif@ksu.edu.sa (A.A.); ahalmogren@ksu.edu.sa (A.S.A.); 2 Computer Science and Engineering Discipline, Khulna University, Khulna, Bangladesh; E-Mail: alamgir@cseku.ac.bd; 3 Ikerlan-IK4 Technological Research Centre, Arrasate-Mondragón 20500, Spain; E-Mail: JParra@ikerlan.es

**Keywords:** entertainment support system, elderly care, elderly entertainment, ambient assisted living

## Abstract

Elderly people constitute a major portion of world's population. Many of them are physically and mentally vulnerable and need continuous support for their health and well-being. There is a growing trend that these elderly people are placed in an ambient assisted living environment (AAL) with an aim to receive better care and support. In such settings, a lot of attention has been given to continuous health monitoring for maintaining physical health status. However, much less attention has been given toward understanding the entertainment needs of the elderly people, which is an important factor relevant to their mental health and joyful living. This paper thus addresses the entertainment needs of the elderly and proposes a framework of an elderly entertainment support system. The proposed framework enables different categories of residents (e.g., elderly people and caregivers) to access various media services in both implicit and explicit manner in order to enhance the quality of their living experience in different contexts. Our experimental results demonstrate the viability of the proposed framework. We believe that the proposed approach will establish the need to develop entertainment systems and services for the elderly people and allow us to sensibly address the problems associated with their independent, happy and active living.

## 1. Introduction

The rapid increase of a worldwide elderly population puts a huge burden on current health and social systems. As a result, a lot of attention has been vested to find novel mechanisms, services and solutions for addressing the health and well-being issues of this elderly population. Many of these elderly people are placed in Ambient Assisted Living (AAL) environments that embed myriad sensors, devices and services to monitor them and provide them relevant and timely services [1,2]. Within the AAL environments, much efforts have been given toward issues of physical health monitoring, although other important issues such as the study of entertainment needs for the elderly people has got less attention [3,4]. Research has shown that the entertainment needs of the elderly people is equally important for their well-being and joyful living [5].

Providing entertainment support and services to the elderly people in AAL environment can improve the quality of their lives while making them more enjoyable [6]. Therefore, it can contribute to the overall health and wellness of the elderly people. Several researches reported that multimedia-enabled entertainment tool can promote effective treatment plan for the elderly people with dementia [5–7]. However, further study is required to obtain a scientific conclusion of the benefits the entertainment facilities can provide from the perspectives of elderly people, caregiver, *etc*. Here, the caregiver may include informal caregivers (family and friends) or formal/professional caregivers, or both.

There are few works that talk about entertainment for the elderly people, such as [6–8], although these works do not provide any framework for entertainment support system based on the needs of the elderly people. The authors in [9,10] studied elderly users' requirements to provide them ICT-based health and care services, including entertainment and information access. However, the goal of these studies was not to develop an entertainment support system for the elderly people but to emphasize that such a system should be based on identifying their requirements and not vice versa. Other existing works focus on elderly monitoring from different perspectives, which include robotic assistance [11,12], reminding service [13], information access [14,15], health monitoring [16–18], and so on. Therefore, despite several works on supporting elderly population, there is a clear need to specifically focus on identifying entertainment requirements for the elderly people and develop an entertainment system framework to incorporate these requirements.

Our contribution in this paper is two-fold. Firstly, we identify the requirements of an elderly entertainment support system from the perspective of different category of residents in AAL environment. Secondly, we propose a framework of an elderly entertainment support system to show how the elderly people can benefit from this framework for accessing different media via different modes of interaction and entertainment delivery channels. The preliminary idea of this work was first reported in [4], which has now been extended with greater detail of the methodology and experiment in this version.

The remainder of this paper is organized in several sections as follows: the related works appear in Section 2, while the proposed elderly entertainment support system is elaborated in Section 3 that includes the description of use cases, object model, interaction paradigm and algorithm, context identification, rule generation, media recommendation, and content selection. Section 4 describes the implementation of the framework and reports the results. A conclusion and future work directions are given in Section 5.

## 2. Related Work

In this section we provide a highlight of the few existing works that study the elderly entertainment issues. Researchers [9,10] have attempted to understand the need for entertainment and information access by the elderly. In [9], user requirements of five different aspects namely information access, communication, self-care, accessibility and personalization of services were identified. Among these, information access included applications such as personal photos, simple games, on-demand TV, radio and video services. The study also identified how likely the older people would use the different information services.

Researchers in [7] described the use of entertainment robot that responds to spoken commands from the people with severe dementia. The authors attempted to persuade that robot-based approach can serve as an effective rehabilitation tool for this group of people. Hilta and Lipschultza [3] studied the behavior of elderly American people and found out that they like to use Internet-based entertainment facilities. The authors suggested the use of radio/television websites to access information and entertainment support for the elderly people. This group of people considers mass media consumption as their main leisure activity and authors in [19] have observed the importance of the usage of media to the elders. N. Alm *et al.* [5,6] have worked with elderly people suffering from dementia, and proposed special interactive virtual environment as an entertainment that can be used without caregivers help.

An artificial companion named “Homie” was proposed by Kriglstein and Wallner [20] as an entertainment and health assistant. For vision impaired people, “Homie” is able to read out text messages. With a certain command “Homie” can record notes and appointments. It can also be used as a remote control for the television based on speech command. Among many other things, Homie can further receive text messages from doctors related to medical information and consultation dates. The Age Invaders (AI) game that is proposed in [21] allows elderly people to play with the young in a physical space and enable parents to participate remotely as well. It does not restrict the players in the physical space to be in front of the computers, allowing them to be spatially free and relaxing.

Researchers have also worked on building architectural framework [22,23], which facilitates the development of flexible elderly care services. Another group has established CareLab [2] at the Philips High Tech Campus in Eindhoven, the Netherlands, which is a one-bedroom hi-tech apartment as a senior-care facility. It is equipped with myriad sensors and devices to study different context situations in which the elderly people will use several applications related to their health and well-being. For example, the Lifestyle Assistant as a remote monitoring service, Cognitive Simulator as a IPTV platform to combine multiple services and functionalities, and Awareness System to maintain surrounding awareness.

Emile Aarts in [24] emphasized that multimedia will play a special role in the ambient intelligence (AmI) era, which would obviously contribute to elder care, as one of the goals of AmI is to support people, and elderly people are the ones who deserve most support from any technological advancement. Authors in [25] explored the potential of AmI in the context of our homes and explained how it can facilitate our lives by providing various services, such as entertainment, device control *etc.*, which would be beneficial to the residents, especially to the elderly people. E. Hollywood *et al.* [26] created an artificial companion that is engaging and entertaining to the elderly people at the same time. The OLDES project [27], as realized in Italy and Czech Republic, provides entertainment services to the elderly people through different channels and animator-based special interest forums.

The authors in [14] described AVANTI project, which facilitates the adaptation of web content and user interface for individuals including elderly people. The Age Invaders (AI) game that is proposed in [21] allows elderly people to play with the young in a physical space and parents to participants remotely as well. It also does not restrict the players in the physical space to be in front of the computers, allowing them to be spatially free and relaxing.

The work reported in [28] focused on developing in-home assistance application for the elderly people. This work, also referred to as case-driven AMI system, aims at sensing, predicting, reasoning, and acting in response to the elderly activities of daily living at home. The authors developed a C-AmI system architecture by synthesizing various sensors, activity recognition, case-based reasoning (CBR), and elderly people in-home assistance customized knowledge.

Several other research have focused on elderly care in the home environment. E. Hollywood *et al.* [26] created an artificial companion called Program Alleviating Loneliness (PAL) for the home-bound elderly. The system is engaging and entertaining to be elderly people at the same time. The OLDES project [27], as realized in Italy and Czech Republic, provides entertainment services to the elderly people through different channels and animator-based special interest forums. Another work with a broader perspective has been reported as iCare [15], which addresses the social and behavioral aspects of aging services. The system supports access to ambient services, unbound information reachability and seamless social connection through the iCare home portal.

The works described above either support or promote entertainment-based elderly care. However, unlike those the proposed work focuses on considering the entertainment needs of the elderly people in various contexts and accordingly describes the development of an elderly entertainment support system for the elderly people.

## 3. Proposed Context-Aware Elderly Entertainment Support System

The proposed system is described in this section with respect to its requirements, object model, system behaviour and several distinctive issues related to the the overall system.

### 3.1. Requirements Specification

**Figure 1.**
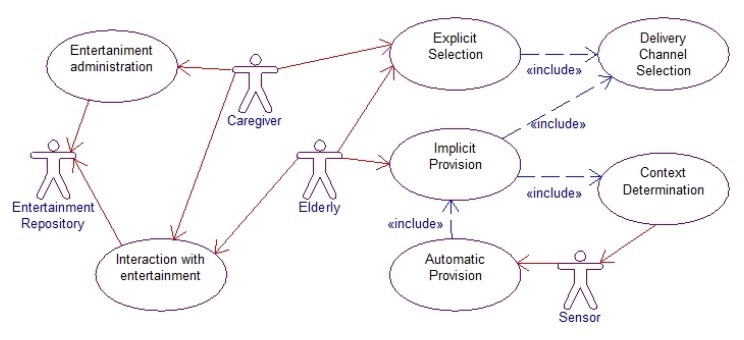
Use case view of the proposed entertainment support system for the elderly people. Several use cases constitute the high-level functionality of the system with which the actors interact.

The primary goal of the proposed system is to provide a flexible mechanism to access entertainment services by the elderly people. In order to do this we resort to the study conducted in [9] to identify the different types of media the elderly people will likely use. We also conducted a field study to identify entertainment needs of the elderly people in terms of required media and interaction and accordingly develop a technical solution for accessing the entertainment media. The specific requirements of the proposed system is presented using a use case diagram [4] as shown in Figure 1. It clearly shows that the caregiver and elderly people are the two main actors of the system who perform the most interactions to obtain entertainment support. From the system point of view, there are two other actors such as Sensor and Entertainment Repository that interact with the system actively or passively. The Sensor actor provides sensory feeds to the Context Determination use case. The Entertainment Repository acts as a passive actor, which provides on-demand access to the entertainment media.

There are several use cases that constitute the high-level functionality of the system with which the actors interact. The three core use cases are Explicit Selection, Implicit Provision and Automatic Provision. These also signify three different types of interactions the elderly people and the caregivers are allowed to perform [29].

The Explicit Selection use case represents the explicit mode of interaction with the system. It provides the option to the elderly people and caregiver to use the system interfaces for identifying a particular entertainment media they would like to experience. Besides selecting the media, the actors in this mode need to select the entertainment channel to express where and how the entertainment is to be provided.

The Implicit Provision use case refers to the implicit mode of interaction through which the actors just mention their interest to access media without specifying any particular media types. The system takes care of the access of relevant media files depending on the current context information. The system also takes care of selecting the entertainment channel for media delivery.

The third mode of interaction is reflected in the Automatic Provision use case, which is initiated by context data. This is different than the other two modes of interaction that are initiated by either the elderly people or the caregiver. Through the Automatic Provision use case, the system automatically selects the relevant entertainment media for the elderly people based on their context and delivers the media through the appropriate delivery channel.

The other use cases of the system are Entertainment administration, Interaction with entertainment, Context determination and Delivery channel selection. While the elderly people and caregiver actors connect to the Interaction with entertainment use case to obtain the media, the Entertainment administration use case will provide functionality for the caregiver. The Context determination use case is not directly used by the elderly people or the caregiver; rather it is initiated by the Sensor actor. Similarly, no actors directly interact with the Delivery channel selection use case, rather this use case is used by the Explicit Selection and Implicit Provision use case.

### 3.2. Object Model

**Figure 2.**
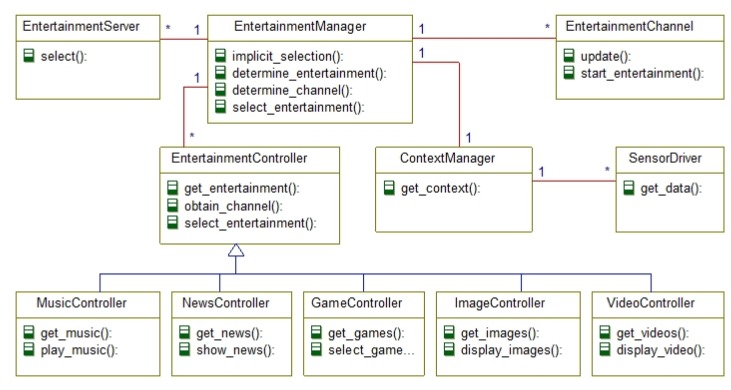
The object model of the proposed elderly entertainment system.

Figure 2 shows the object model representing the use case view of the entertainment support system. This also shows the multiplicity of the connected objects. The EntertainmentManager is the core object of the system, which is connected to many EntertainmentServer and EntertainmentChannel, as in reality there could be many of these objects using which the system can obtain entertainment and deliver it via different channels. Similarly, several EntertainmentControllers may be connected to the EntertainmentManager object with the rational that the elderly and the caregiver may adopt different ways to interact with the system, and the individual EntertainmentController would facilitate that. For example, the elderly may use a different hardware interface to interact with the system than that of the caregiver, which will require different EntertainmentController to accommodate this variation.

The MusicController, NewsController, GameController, ImageController, and VideoController are the various specializations of EntertainmentController to access the different types of media. For example, the VideoController controls which video need to play and how to render the video in a display or mobile devices. Similarly, the NewsController is responsible to fetch the news from the Internet; the MusicController performs necessary action to play music or sound; and so on. Besides these, the ContextManager receives sensor data based on the deployed sensors of heterogeneous types and determines the current context. The context information is passed to the EntertainmentManager based on which different modes of interaction gets activated and appropriate entertainment media is selected for the elderly people. It should be noted that Figure 2 does not show the entity objects that hold the persistent or transactional data.

### 3.3. System Behaviour

**Figure 3.**
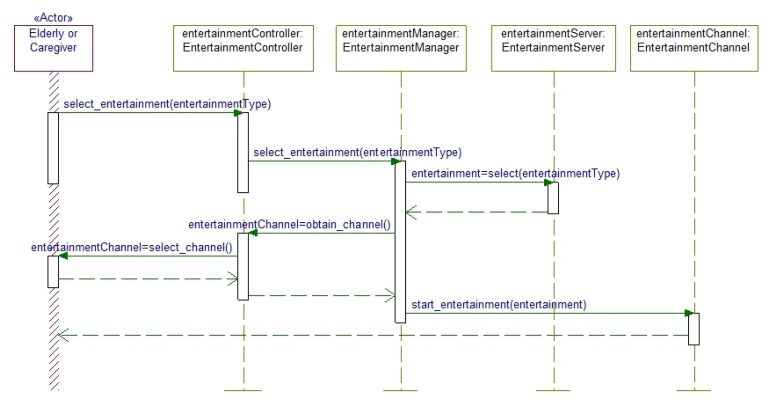
Explicit interaction mode where the elderly or the caregiver initiates the interaction.

The behavior of the proposed system is depicted in terms of several interaction diagrams. For clarity, we mainly focus on the different types of interactions that the user performs with the system as mentioned before. Accordingly, Figure 3 represents the Explicit mode of interaction and its corresponding object interactions in terms of message passing and method invocation. It is visible from this figure that the elderly or the caregiver initiates the interaction. The EntertainmentManager receives the entertainment selection request from the elderly people or the caregiver via the EntertainmentController and fetch the requested media from the EntertainmentServer. The EntertainmentManager also initiates the selection of appropriate entertainment channel through the EntertainmentController, which obtains the channel selection from the elderly person or caregiver. The EntertainmentManager then delivers the selected entertainment based on the selected channel.

**Figure 4.**
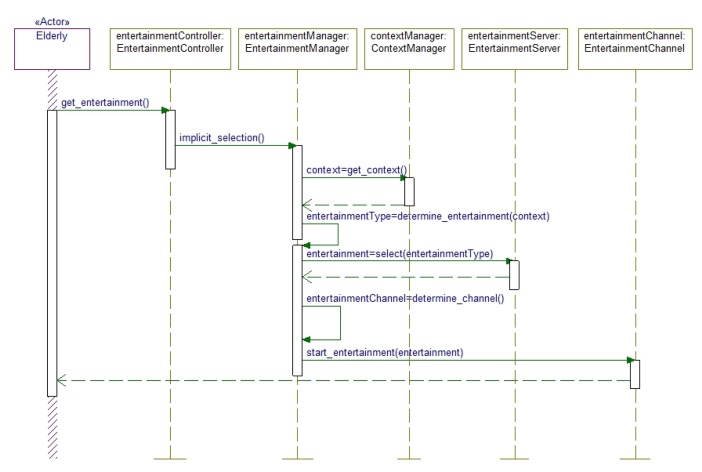
Implicit provision mode where the user's entertainment request is passed to the EntertainmentManager through EntertainmentController.

The Implicit mode of interaction is expressed in Figure 4. In this mode, the user's intention to consume entertainment media is passed to the EntertainmentController object, which contacts the EntertainmentManager to fulfill the user's request. The EntertainmentManager then consults the ContextManager to get the current context and based on this context information entertainment media type and appropriate media are fetched for the elderly people. The context is determined by using different sensor data. Based on the context parameters the system selects the entertainment channel and delivers the media.



**Figure 5.**
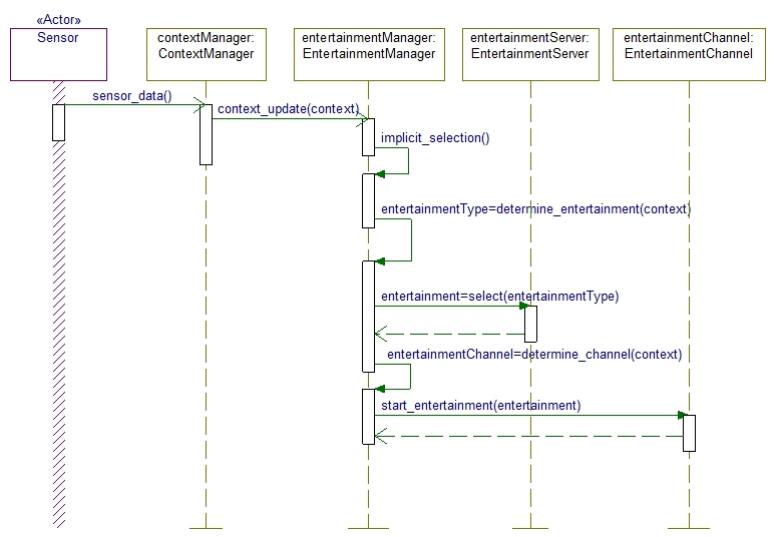
Automatic provision where the ContextManager trigger the particular activity when it receives specific data from the sensors.

**Algorithm 1:** Activity monitoring
 **Input:** An inactivity threshold: *ζ* **while**
*(continuous sensing of the assisted living environment for entertainment)*
**do**  **if**
*(elderly*/*targeted person present)*
**then**   **while**
*(continuous sensing of current context)*
**do**    *ψ* ← get_context();    **if**
*(command or gesture for inactivity)*
**then**     programInactivity ← 1;     //No automatic decision during relaxation or sleep    **if**
*(command or gesture for activity)*
**then**     programInactivity ← 0;    **while**
*(programInactivity*=*0)*
**do**     **if**
*(ζ minute of inactivity by elderly)*
**then**      ϒ ← get_data(*ψ*);      **if**
*(permissionCaregiver(*ϒ*)*= *‘ok’)*
**then**       p ← permissionElderly(ϒ);       **if**
*(p =‘ok’)*
**then**        startMedia(ϒ);       **if**
*(p = ‘cancel’)*
**then**        abortMedia();       **if**
*(p = ‘notNow’)*
**then**        inactivityCounting(Begin);      **else**       //Do nothing. Loop will generate new suggestion     **if**
*(targeted person commands to bring media)*
**then**      op ← determine_entertainment(*ψ*);      start_entertainment(op, get_channel());      **if**
*(Caregiver commands to bring Media)*
**then**      op ← determine_entertainment(*ψ*);      p= permissionFromPerson(op); start_entertainment(op, p, get_channel());      informCareGiver();  **else**   programInactivity ← 1;


The other system behavior is presented in Figure 5 to highlight the Automatic provision mode of interaction. In this mode, the system's behavior is initiated by the Sensor from which data passes to the ContextManager to identify the current context. This mode involves the object interaction among the Sensors (as actors), ContextManager, EntertainmentManager, EntertainmentServer, and EntertainmentChannel. The EntertainmentManager starts the entertainment media with the help of the EntertainmentServer and EntertainmentChannel.

The proposed entertainment support system continuously senses the environment, context, time, motion of automated home/room as per the Algorithm 1. Firstly it works only when the targeted person is present in the assisted living environment. The person is identified by tags and sensors. The program can also identify caregiver. After ensuring the existence of elderly it starts the activity based on the three modes of interaction just described.

The program can detect inactivity of a person. This inactivity is determined based on his motion, his position, his commands, inactivity in program (no media is played for a certain time). After detecting inactivity for a certain time (we fixed it with 30 minutes, it is adjustable by the caregiver) the program starts to make decisions. First, it calls a portion of program that is responsible for generating suggestion from the available media. We will discuss this program portion in detail later. Generating a suggestion cause the program to ask for permission from caregiver. Then caregiver determines if the selection is ok and sends ‘OK’ command which lets the program to ask permission from the targeted person himself. The person can accept it with “OK”, reject it with ‘CANCEL’ or can postpone the whole entertainment procedure with ‘NOT-NOW’. If system gets “OK”, it starts the media. If it gets “CANCEL”, it cancels the current suggestion and generates another one. If it gets “NOT-NOW” it restarts the whole procedure, that means it waits for another 30 minutes to see if inactivity level changes.

A person can anytime select his entertainment media and can control it via explicit interaction. This mode is straight forward. If he gives the command to start media, the program will present options before him. These commands can be given through voice/gesture/button press. After the selection of options, the program will start the media. There is no need for the presence of the caregiver in this mode.

There could be disabled people who cannot take decisions or commands for themselves. A caregiver can take decisions for them at that moment or if a caregiver thinks it is necessary he could give the command to start entertainment media. At his command the program will generate a suggestion. If the caregiver approves, this suggestion will be presented to the person and then follow the same procedure that we discussed on the first mode. The person can send ‘OK’, ‘CANCEL’, ‘NOT-NOW’ signal with command or gesture, *etc*.

### 3.4. Context Awareness

The proposed system adopts context-aware approaches to select and deliver media to the elderly people. Whether or not it is context-aware or situation aware depends on the ContextManager that receives sensory feeds from heterogeneous sensors and processes it to determine the current context. User context depends on different factors like user's current mood, appearance, activity, *etc*. The environment contexts also influence the final service like current temperature, days of the week, room lighting condition, *etc*. Analyzing different sensor data and accordingly extracting the current context is a complex process and depends on different vision and resonance technologies like face detection, face recognition, gait recognition, video fusion, affection computation, *etc*. For the context analysis we consider the popular software process and projects questionnaire called *W*^5^*HH* [30] which is a series of questions that lead to a definition of key software project characteristics and resultant project plan. We consider those questionnaires to extract the characteristics of a particular user context. For user context determination the first important thing is what type of job or activity the user is currently doing like sleeping, reading, playing, *etc*. Based on his/her present activity the context semantic will be different.

The next important question for context calculation is when the user performing the job. Based on the time of activity like morning, evening, on Sunday, on his birthday the context value will be different. User current job place has also important for context analysis. If the user is currently in his reading room and is reading a book then he may not like to listen to music on the audio system, whereas if he is currently in the living room and reading a book then maybe he likes to listen some soft music on the audio system. So where the jobs are being performed is an important factor for user context analysis. The next question is who is responsible for which job. We modified this question little bit and decide the person who is responsible for the current job and who is accompanying with him or her. For example, if an elderly person is with another elderly person or caregiver, the system might select some media that are different than when the elderly person is alone in a place.

User's current mood is also an important factor for context calculation. If a user is happy he/she can listen any type of music but when he is in sad mood he might expect some sad music. The job duration is also an important factor. For example, if a user has been reading for the last two hours, his expectation from the system might be different than reading for 10 minutes. After reading a long time people got tired and need to relax like watching favorite movies or listening to news headlines. On the other hand, reading for a short period is not tiring and hence the activities requiring brain work can be done. Finally, the resource utilization of how much of the environment resources will be utilized is a factor for the context analysis. For example, users need low consumption of power for the light, heating system, air condition system, so based on his/her interaction the context may be different. Practically, a perfect user context determination can rarely be achieved, although the *W*^5^*HH* can fairly determine the current user context if sufficient data is provided. Table 1 demonstrate this *W*^5^*HH* context determination questions. The total user context value is calculated from this seven question by using Equation (1), where *W* and *H* are those seven question values.

(1) $$C_{x}=\prod_{i=1}^{5}W_{i}\times \prod_{j=1}^{2}H_{j}$$

In different contexts, the elderly people may need different support with respect to entertainment needs. For example, an elderly people may need to get the updated news about what is happening in certain places before going to sleep. Context identification is a fairly independent process that continuously takes place, and other system objects that needs this context information, can access such information to act or react.

### 3.5. Rule Definition

Defining appropriate rules to select appropriate entertainment for the elderly is important. This could be simple if-then-else rules or more formal rules like Event Condition Action (ECA) [31] that defines rules based on events. The fundamental construct of ECA is reactive rules such as, *On* <*event expression*>, *If* <*condition*>, *Do* <*action*>. This means that *on* detecting certain events (*i.e.*, elderly activity), *if* certain conditions are verified (*i.e.*, true), then specific actions should be executed (*i.e.*, *do*). More formally, ECA is a short-cut to represent the structure of active rules especially for event-driven architectures and active database systems. ECA languages are indeed an intuitive and powerful model of programming that deals with reactive systems.

Based on the foregoing ECA rule construct, we can model the cases defined in Section 3.1 as the following:

**On** elderly person laying on the couch in the afternoon
**If** the elderly person is not sleeping
**Do** provide soft music.

### 3.6. Entertainment Media Recommendation

In order to provide relevant and enjoyable entertainment media, the system needs to learn the behaviour of the elderly people over a period of time. There are many different techniques to do so ranging from data mining to user modeling to recommender system approaches. One of our earlier works focuses on this by considering the past user interactions with the environment, user profile and media reputation to select the best media services for the people [32,33]. Such an approach is reasonably applicable in the case of the proposed framework. However, it is imperative that preliminary interview is conducted with the real elderly people for whom the proposed system is targeted. Through such interviews, it is possible to get the information about the type of media the elderly people prefers, the time when media is appreciated and the media delivery channel. It is worth mentioning that the entertainment does not only mean to access movies, images, or animation, the process of accessing these media should be enjoyable.

In this paper, we have defined the media recommendation or suggestion generation rules and based on them the system suggests which media can be played at any moment. Though many types of media can be selected we have shortlisted six types of entertainment media for our framework as per the initial field study and existing user study such as in [9,10]. The program and/or users can select any option from these six types of media: Movie, Music, Game, News, Image, and Drawings. We have defined three types of scoring, based on which appropriate media will be selected. These scoring are MomentScore, PriorScore and UsedScore.

#### 3.6.1. Moment score, *η*

Moment score is the context score which is calculated by different environmental factors like user mood, time of the day, user profile data, *etc*. In our proposed approach the whole day is divided into 4 time segments and a person's context is divided as per 4 different moods (e.g. energetic. sleepy, relaxing and bored). Then the points for mood and time of the day are divided based on the caregiver's judgment and selection.Table 2 shows this point distribution. The moods are determined by activity, position, command, sensors, *etc*. Total score of each cell is normalized to one. Here, larger number of moment score means more priority for a combination.

#### 3.6.2. Prior Score, *θ*

Prior score is the initial score of a person for different media. Prior score is set based on the choice of the person himself. This value helps a person to personalize the media priority. He can assign priority at the beginning of the program execution (also can update the initial score at any time). For example, if he wants to see more movies than hearing music then the person needs to assign more points to movie than music. Table 3 shows the Point score of a person. It should be noted here that for every person the total point score for different media must be equal to one. So whenever the person will set an initial value for a specific media then the other values will be adjusted so that the total value should be equal to one.

#### 3.6.3. Used Score, *ζ*

Used score or profile score is calculated based on how much a person already used a media selection. This score is important; as for media selection the media values will be adjusted so that the other media that has less priority can be selected. In other words it shuffles the media so that the system can play different media rather than playing same media again and again. Initially the system assigns equal values to all media. When a person used a particular media, the used score for that particular media decreases and decrement value is divided equally between other media options. Initially five types of media options have used score as 0.20. Table 4 shows the change of used score after each media selection.

So after each selection, 0.8 is deducted from the selected media, which is divided equally (0.2) among other options. At all-time, the summation of the total Used score is 1. The media option with the most total score (Equation(2)) gets selected.

(2) Totalscore=η+θ+ζ

### 3.7. Content Selection

The recommended media can be of different type, such as Movie, Music, Games, News, Image, and Drwaing. In the following, we are going to discuss the content selection for each media option.

#### 3.7.1. Movie

Movie is considered as part of the entertainment media for the elderly people [3]. We consider 4 genre while selecting movie, which are Romance, Drama, Thriller, and Action. In order for a movie to be selected, the Prior score and Used score for movie genre (Equation(3)) are used. Tables 5 and 6 shows the interaction of the movie genre selection with each encounter. Once a genre is selected a random movie gets selected from that genre. These movies are predefined by the caregiver.

(3) Total score for a genre=Priorscore+Usedscore

#### 3.7.2. Music

Music is an important entertainment media for elderly people [34,35]. In the music selection the system considers different factors like music genres (Romantic, Classical, Old decades, Pop), time of the day, the users profile data, elderly people or caregivers choices, *etc*. Like the video genre the music genre has an initial score which will be updated based on the users preferences or media playback. The time of the day is an important factor for music play. Normally elderly people like to hear music at the morning and at the evening; they do not like to hear music at late night. So based on this myth the system explicitly or automatically selects music.

#### 3.7.3. Games

Games are selected based on the physical ability of each person [21]. There are some games defined by the caregiver based on the ability of the person. Suppose a person's hands are weak, he can play with his voice. So a game has to be selected for him from android market as an example, which can be played through voice. Or voice-impaired people can play with eye gesture or hands. We have selected few games which are assigned by the caregiver to a particular person based on his ability. Games like Chess, Mahjong Titans, Cards and Need are played for speed. If several games are defined for a user, a game is randomly selected at the time of suggestion generation.

#### 3.7.4. News

News is an important entertainment media for the elderly [3,10]. If news media is selected by explicitly or automatically then the program brings news from the Internet. In this case the entertainment repository server fetches the latest news from different news providers like yahoo, BBC, CNN, *etc*. It receives raw text from different web servers by HTTP request and finally based on the keyword matching it prepares a news rank list and presents the top news to the user's display or the mobile devices. It should be noted here that the old people or the caregiver can set the number of top news that the program should display or they can add news related keywords so that the system can filter appropriate news. This is important because sometimes the old people might not like political news or the violent news, for example. Therefore, the user can add the negative keywords to the system so that it removes all the news that matches with the negative keyword from the news list.

#### 3.7.5. Image sliding

User studies have identified images as one of the preferred media of choices [9]. Recalling historical moments using image sliding may bring comfort to the elderly people. When the system detects any events like birthday, anniversary or any special day like international mothers' / fathers' day, victory day, *etc.*, then the system fetches the images from the entertainment repository. If sufficient number of images not found in the repository, the system fetches images from the Internet and finally starts the image slide show in the user's mobile or display devices.

#### 3.7.6. Drawing

Often, drawing can be entertaining for the elderly people as we identified in our field study. Normally, elderly people want to draw something by using simple tools. Digital drawing, like a simple paint brush application or drawing pad could be a nice solution where elderly people can draw anything and can share his/her works with others by using the Internet.

#### 3.7.7. Media rendering

Whether the entertainment is to be delivered based on different modes of interaction, it is important that the selected entertainment media is rendered appropriately, which should satisfy the elderly. For this to be realized, an assisted living environment should have some sort of mechanism to control different entertainment devices through intuitive interfaces, such as NFC-enabled touch [36].

## 4. Implementation and Result

This section describes the implementation details of the prototype that reflects the proposed elderly entertainment support system presented in Section 3. The prototype integrates the main three modes of interaction, *i.e.*, implicit interaction, explicit interaction and automated choices. The prototype performs the functionality of the automated suggestion of entertainment media and resources as well as controls the whole environment checking like context reading, current mood, choices made by people and caregivers. Microsoft *C*# version 8.0 and the SQL server 2005 database server were used to develop the prototype system. In the following, Section 4.1 describes the experimental setup and the different devices that are connected within the environment, while Section 4.2 briefly discusses the entertainment selection and media suggestion process.

### 4.1. Environment Setup

**Figure 6.**
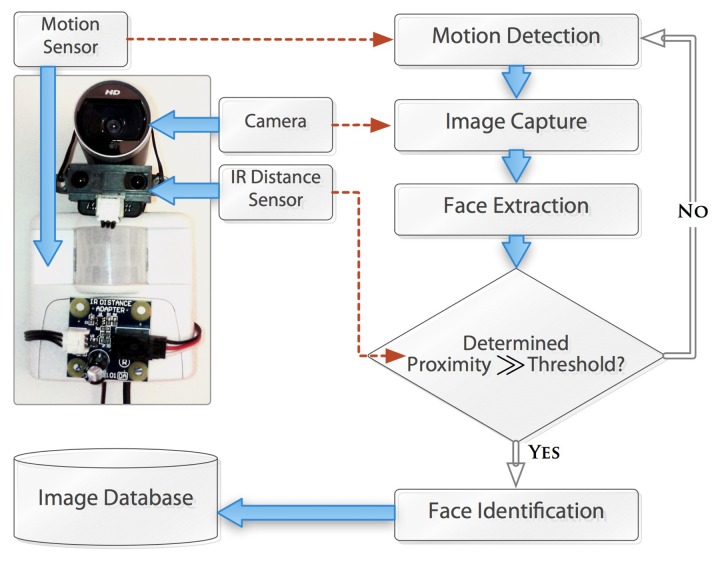
Camera and sensory device combos that were installed in four different locations in the test environment.

This section describes the experimental setup that we conducted in our laboratory environment. The motivation of our experiment was to produce a data set so that we can work on the dataset to determine the performance of our proposed approach. Figure 6 shows the camera and sensory device combos that were installed in four different locations in the test environment. For the real-time monitoring, we used high definition Microsoft *LifeCam* 3.0 camera, *X*10 *Pro Occupancy* motion sensor and *Sharp IR* distance sensor. The camera had built-in microphone and we utilized that to capture the sound. The prototype system took images periodically with certain triggered events. The events were triggered by monitoring the microphone sound as well as the motion tracking IR devices.

For the validation of our Algorithm 1 the prototype system stored the captured images into the disk through the SQL server database and recognize the face using [37]. Sensors are used for detecting weather outside, persons presence in room, or positions like sitting, walking. To present suggestions for permission we used several mechanisms so that people with different disabilities can get the media suggestions. For example, people with low eyesight are presented with suggestions by sounds and their confirmation is taken with voice commands. On the other hand, people with hearing problems see suggestions visually on screen and gave command through voice or buttons, *etc*. Caregivers also used voice commands.

### 4.2. Results

The goal of our evaluations is to determine the performance of our elderly entertainment support system. For this reason, we invited four male and four female subjects to participate in our experiments. These people were aged between 60 years to 70 years. They had different kind of disabilities: two persons have low eyesight and one has a hearing problem. This is why we installed different types of sensors and mechanisms to communicate with them. These old persons were found through local old age home and known people. We also provided four more known professional people (three female and one male) as caregivers who were with them the whole time. We set up an automated home with sensors and program as described in proposed architecture and implementation section. Each subject stayed for four days in our experimental room. For complexity reduction the old person stayed with the system only at day time from 8 am to 9 pm each day. Based on those experiments we gathered some data. The overall result is divided in three categories. The first is the performance of our prototype based on the acceptance and rejection of suggestions. The second one is based on which kind of entertainment media is more preferable to them. The last one is the qualitative study.

#### 4.2.1. Preferable Media for Persons (PriorScore Calculation)

**Figure 7.**
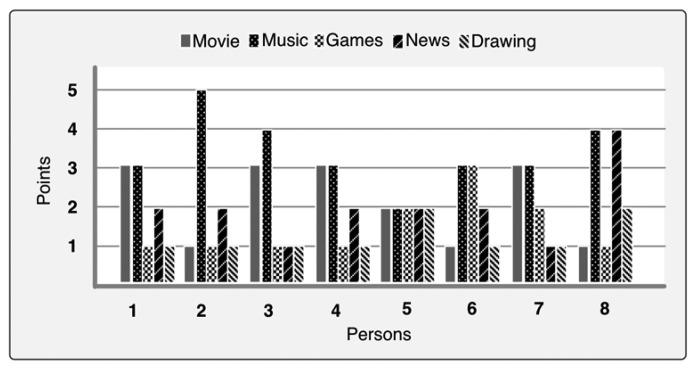
Preferable media for persons.

**Figure 8.**
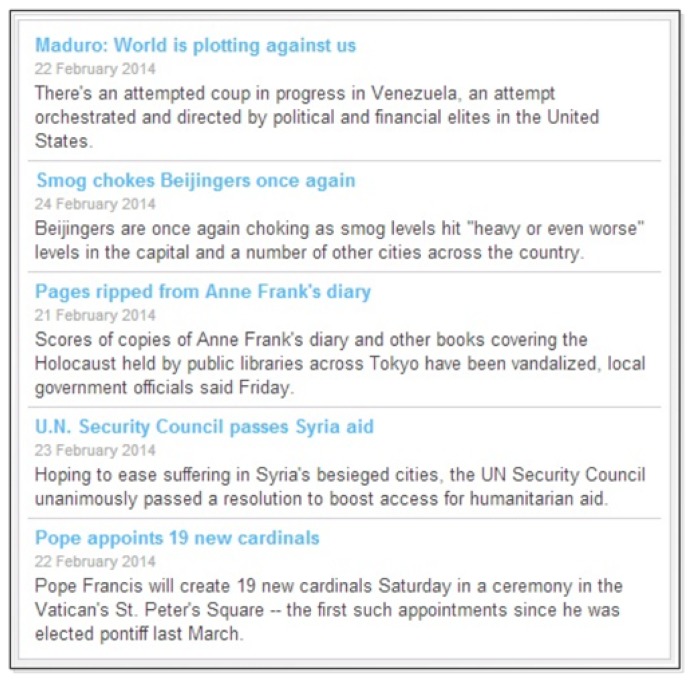
For news, we built a web service that brings data from different RSS feeds.

At the beginning of experiment we asked old people their choices for the media entertainment. We presented them with five media entertainment options as we mentioned in the prototype. We said to give points to each media out of 10. They had to distribute their points as a series such that their most preferable media gets the highest score and so on. Based on Figure 7, we can see that most preferable media is music. It got 32.5%. Then movie got 21%, news got 18.7%, games got 15% and drawings 12.5%. We calculated PriorScore with this result by dividing each media point by 10 and used in the implementation of our prototype. This result is very important to determine which media is more preferable to whom. Figure 8 shows a snapshot of RSS news feeds that were brought using web service.

#### 4.2.2. Performance of the Suggestion Generation

**Figure 9.**
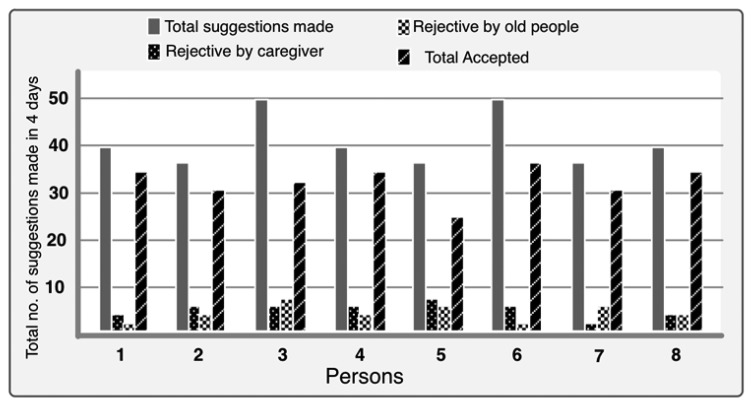
Performance of the suggestion generation.

For the purpose of measuring the performance of our prototype we calculated the total acceptance and rejection by the caregivers and old persons. We summed all the suggestions made for a person in 4 days and noted the rejected and accepted numbers. Figure 9 shows the results of these criteria. For the first person, a total of 41 suggestions were made by our prototype and the caregiver did not allow 4 media entertainment suggestions because he did not feel appropriate to provide the media to old person at that moment. Two suggestions were rejected by the person himself because of he did not like the content and was not in the mood for that content at that moment. Thirty five suggestions were successfully allowed and those contents were provided to that person. In Figure 9, the horizontal axis represents each person and vertical axis represents the total number of suggestions in 4 days for each person. Based on this data we find that 78.5% of suggestions were accepted by both caregivers and old persons whereas caregivers' and persons' rejection rates are around 11%.

#### 4.2.3. Qualitative Study

**Figure 10.**
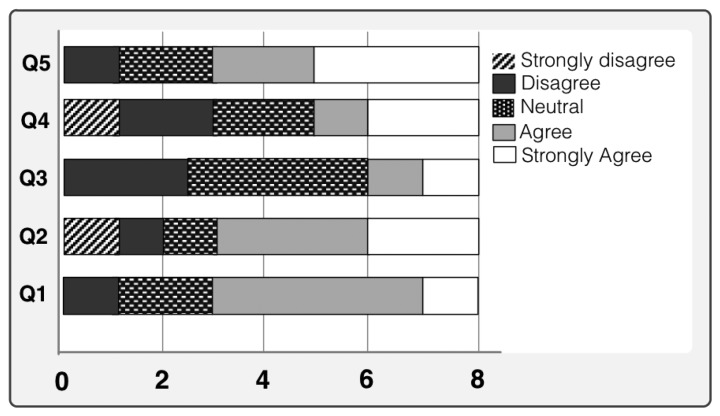
Old user's responses for each given assertions that are listed in Table 7.

**Figure 11.**
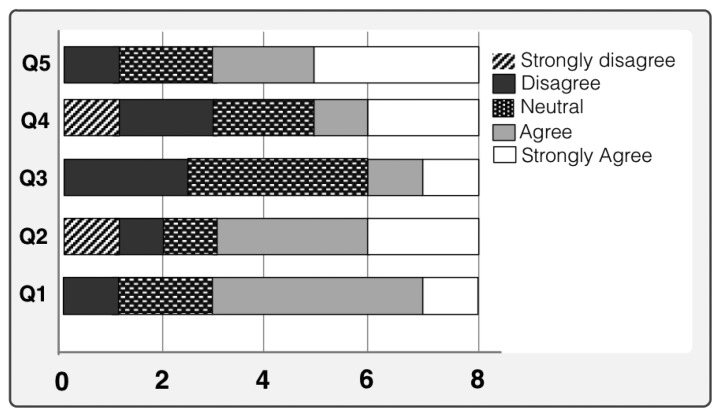
Caregiver feedback based on the questioner listed in Table 8.

To evaluate the user's quality of experience with the prototype and to justify the suitability of our proposed approach we have performed few qualitative measurement studies. The qualitative analysis is performed by studying different usability aspects of the proposed system. We have determined different usability aspects of our prototype and designed our tests accordingly. Before performing the usability test we designed a test plan where we defined our evaluation objectives and developed questions for the participants. Two different type of qualitative measurements are performed for both old persons and caregivers. As they both used the system and complement each other, it was necessary to evaluate prototype from both perspectives. Therefore, we presented two different sets of questionnaire to respective persons and collected their answers. The user responses are shown in Likert Scale [38] in Figures 10 and 11. The ratings of the questionnaire were in the range of 1–5 (the higher the rating, the greater was the satisfaction). The average of the responses of the users were calculated in percentage form and measured after the usability tests.

Figure 10 shows the user's responses for each given assertions. It is worth mentioning that more than 70% of the old users would like to use the system. Overall, 65% of the old users were also satisfied with the automatic environment including sensors. Nearly 80% are happy to use this prototype and enjoyed this service. Finally, Figure 11 shows the picture of caregivers' acceptance of system based on the questions asked. Three out of four caregivers happily accepted the system. They all believe that this system makes their clients happier and entertained them.

## 5. Conclusions

This paper proposes a novel context-aware elderly entertainment support system for the elderly people. It focuses on the design and development of the system by identifying its requirements from the perspective of elderly people and caregivers. Among the notable functionality of this system, it describes explicit, implicit and automatic provision modes of interaction for the elderly people and caregivers. The paper also highlights some of the distinctive issues, such as context awareness, entertainment recommendation, rule definition, media selection, *etc*. We presented the implementation details of a preliminary prototype as per the requirements. Finally, from our usability study we received suggestions from both old people and the caregiver to improve the media suggestion mechanism and add more media types like whether forecasting, highlight memorable events, talking facility with the system, *etc.*. In the future we want to incorporate more sensors in a real assisted living environment to provide intuitive interaction capabilities to the user in order to effectively control the entertainment. We also plan to involve more users in the test in realistic settings.

## Figures and Tables

**Table 1.** *W*^5^*HH* Principle.

What will be done (activity)	Working, Reading, Sleeping
When will be done	Weekday, Weekend, Morning, Evening
Where will be done	Indoor, Outdoor, Home, Office, Bed Room
Who is responsible (with whom)	Parents, Friends, Children, Family, Alone
Why (in what mood)	Happy, Sad, Anger, Grief
How the job will be done (how long)	Ten minutes, one hour
How much of each resource is needed	Light, Volume, Air Conditioning

**Table 2.** Point distribution based on the moods and time of the day.

	7.00 am	12.00 pm	4.00 pm	7.00 pm	–12.00 pm	–4.00 pm	–7.00 pm	–12.00 am

Energetic	Mo-0.2	Mu-0.4	Mo-0.4	Mu-0.05	Mo-0.2	Mu-0.05	Mo-0.2	Mu-0.3
	Ga-0.1	Ne-0.2	Ga-0.05	Ne-0.2	Ga-0.4	Ne-0.05	Ga-0.025	Ne-0.4
	Dr-0.1		Dr-0.3		Dr-0.3		Dr-0.075	

Sleepy	Mo- Mu- Ga- Ne-Dr-		Mo- Mu- Ga- Ne-Dr-		Mo- Mu- Ga- Ne-Dr-		Mo- Mu- Ga- Ne-Dr-	

Relaxing	Mo- Mu- Ga- Ne-Dr-		Mo- Mu- Ga- Ne-Dr-		Mo- Mu- Ga- Ne-Dr-		Mo- Mu- Ga- Ne-Dr-	

Bored	Mo- Mu- Ga- Ne-Dr-		Mo- Mu- Ga- Ne-Dr-		Mo- Mu- Ga- Ne-Dr-		Mo- Mu- Ga- Ne-Dr-	

Mo- Movie, Mu-Music, Ga- Games, Ne- News, Dr- Drawing.

**Table 3.** Prior score of a person for different media.

	Movie	Music	Games	News	Drawings
Person 1	0.3	0.3	0.1	0.2	0.1

**Table 4.** Used score and its changes after each media selection.

	Movie	Music	Games	News	Drawings
Initial score	0.20	0.20	0.20	0.20	0.20
After 1st selection as movie	0.12	0.22	0.22	0.22	0.22
After 2nd selection as Music	0.14	0.14	0.24	0.24	0.24

**Table 5.** Prior score of a person for different movie genres.

	Romance	Drama	Thriller	Action
Person 1	0.4	0.4	0.1	0.1

**Table 6.** User score for one person for different movie genres.

	Romance	Drama	Thriller	Action
Initial score	0.25	0.25	0.25	0.25
After 1st selection as Romance	0.10	0.20	0.20	0.20

**Table 7.** Questions: (For old people).

Q1	Was it easy to get used to with the automatic home environment including sensors?
Q2	Are you happy with the overall entertainment media selection for you?
Q3	Do you think more media option is required?
Q4	Are you satisfied with the time-interval between two suggestion generations?
Q5	Do you feel better than before?

**Table 8.** Questions: (For Caregiver).

Q1	Does it help you in taking care of the old people?
Q2	Are you overall happy with the entertainment media selection or have you happily accepted the system?
Q3	Do you feel persons under your supervision are more happier than before?
Q4	Are you satisfied with the time-interval between two suggestion generations?

## References

[1] Sun H., De Florio V., Gui N., Blondia C. Promises and challenges of ambient assisted living systems.

[2] De Ruyter B., Pelgrim E. (2007). Ambient Assisted-Living Research in CareLab. Interact.

[3] Hilt M.L., Lipschultz J.H. (2004). Elderly Americans and the Internet: E-mail, TV news, information and entertainment websites. Educ. Gerontol..

[4] Hossain M.A., Alamri A., Parra J. Context-aware elderly entertainment support system in assisted living environment.

[5] Alm N., Astell A., Gowans G., Dye R., Ellis M., Vaughan P., Riley P. (2009). Engaging multimedia leisure for people with dementia. Gerontechnology.

[6] Alm N., Astell A., Gowans G., Dye R., Ellis M., Vaughan P., Newell A.F. (2007). An interactive entertainment system usable by elderly people with dementia. Universal Access in Human-Computer Interaction. Ambient Interaction.

[7] Tamura T., Yonemitsu S., Itoh A., Oikawa D., Kawakami A., Higashi Y., Fujimooto T., Nakajima K. (2004). Is an entertainment robot useful in the care of elderly people with severe dementia?. J. Gerontol. Ser. A: Biol. Sci. Med. Sci..

[8] Matsuyama Y., Taniyama H., Fujie S., Kobayashi T. System design of group communication activator: An entertainment task for elderly care.

[9] Brownsell S., Blackburn S., Hawley M. (2012). User requirements for an ICT-based system to provide care, support and information access for older people in the community. J. Assist. Technol..

[10] Lehto P. (2013). Interactive CaringTV^®^ supporting elderly living at home. Australasian Med. J..

[11] Montemerlo M., Pineau J., Roy N., Thrun S., Verma V. Experiences with a Mobile Robotic Guide for the Elderly.

[12] Gross H.M., Schroeter C., Mueller S., Volkhardt M., Einhorn E., Bley A., Martin C., Langner T., Merten M. Progress in Developing a Socially Assistive Mobile Home Robot Companion for the Elderly with Mild Cognitive Impairment.

[13] Si H., Kim S., Kawanishi N., Morikawa H. A Context-aware Reminding System for Daily Activities of Dementia Patients.

[14] Finka J., Kobsaa A., Nill A. (1988). Adaptable and adaptive information provision for all users, including disabled and elderly people. N. Rev. Hypermedia Multimed..

[15] Chang W., Yuan S., Li E.Y. (2009). iCare home portal: An extended model of quality aging e-services. Commun. ACM.

[16] Ohta S., Nakamoto H., Shinagawa Y., Tanikawa T. (2002). A health monitoring system for elderly people living alone. J. Telemed. Telecare.

[17] Gupta G.S., Mukhopadhyay S.C., Sutherland M., Demidenko S. Wireless sensor network for selective activity monitoring in a home for the elderly.

[18] Palumbo F., Ullberg J., Štimec A., Furfari F., Karlsson L., Coradeschi S. (2014). Sensor Network Infrastructure for a Home Care Monitoring System. Sensors.

[19] Robinson J.D., Skill T., Turner J.W. (2004). Media usage patterns and portrayals of seniors. Handbook of Communication and Aging Research.

[20] Kriglstein S., Wallner G. (2005). HOMIE: An artificial companion for elderly people. CHI 2005 Extended Abstracts on Human Factors in Computing Systems.

[21] Khoo E.T., Cheok A.D., Nguyen T.H., Pan Z. (1988). Age invaders: Social and physical inter-generational mixed reality family entertainment. Virtual Real.

[22] Raij K., Lehto P. (2008). Caring TV as a service design with and for elderly people. New Directions in Intelligent Interactive Multimedia.

[23] Camarinha-Matos L.M., Afsarmanesh H. (2004). A multi-agent based infrastructure to support virtual communities in elderly care. Int. J. Netw. Virtual Org..

[24] Aarts E. (2004). Ambient Intelligence: A Multimedia Perspective. IEEE MultiMedia.

[25] Friedewald M., Da Costa O., Punie Y., Alahuhta P., Heinonen S. (2005). Perspectives of ambient intelligence in the home environment. Telemat. Inf..

[26] Hollywood E., O'Brien G., Lennon S. SIGCHI project: User centered design of a program alleviating loneliness (PAL).

[27] Busuoli M. Entertainment and ambient: A new OLDESv́iew.

[28] Zhou F., Jiao J., Chen S., Zhang D. (2011). A Case-Driven Ambient Intelligence System for Elderly in-Home Assistance Applications. IEEE Trans. Syst., Man, Cybern.-Part C: Appl. Rev..

[29] Hossain M.A., Ahmed D. (2012). Virtual Caregiver: An Ambient-Aware Elderly Monitoring System. IEEE Trans. Inf. Tech. Biomed..

[30] Boehm B. (1996). Anchoring the Software Process. IEEE Softw..

[31] Liu J., Augusto J., Wang H. (2006). Consideration on uncertain spatio-temporal reasoning in smart home systems.

[32] Hossain M.A., Atrey P.K., El Saddik A. (2008). Gain-based Selection of Ambient Media Services in Pervasive Environments. Springer J. Mob. Netw. Appl..

[33] Hossain M.S., Hossain S.A., Alamri A., Hossain M.A. (2013). Ant-based service selection framework for a smart home monitoring environment. Multimed. Tools Appl. (MTAP).

[34] Kusek D., Leonhard G., Lindsay S.G. (2005). The Future of Music: Manifesto for the Digital Music Revolution.

[35] Laukka P. (2007). Uses of music and psychological well-being among the elderly. J. Happiness Stud..

[36] Iglesias R., Parra J., Cruces C., De Segura N.G. Experiencing NFC-based touch for home healthcare.

[37] OpenCV on Sourceforge. http://sourceforge.net/projects/opencv/.

[38] Likert R. (1932). A Technique for the Measurement of Attitudes. Arch. Psychol..

